# An Act to Establish a Board of Veterinary Medical Examiners in the State of Pennsylvania

**Published:** 1894-09

**Authors:** 


### AN ACT

To Establish a State Board of Veterinary Medical Examiners, and to Regulate the Practice of Veterinary Medicine and Surgery in the State of Pennsylvania.

Section 1. Be it enacted by the Senate and House of Representatives, etc.: That a Commission is hereby established to be known as the State Board of Veterinary Medical Examiners, to consist of five commissioners, who shall be members, in good standing, of the veterinary profession, who shall be graduates from a recognized veterinary college, or colleges, and who shall hold their office until their successors are appointed and duly qualified. Said Board shall have power to adopt by-laws and regulations, such as they may deem advisable to carry into effect the provisions in this Act, provided the said by-laws shall not conflict with the Constitution or laws of this State, or of the United States.

Section 2. Said Board shall consist of five members, and each of the said members shall serve for a term of three years, from the 1st of March next after his appointment, with the exception of those first appointed, who shall serve as follows: One for one year, two for two years, and two for three years, from the first day of March, Anno Domini one thousand eight hundred and ninety-five.

The Governor shall, in his appointments, designate the number of years for which each appointee shall serve. The appointments of successors to those members whose term of office will expire on the first day of March of each year, shall be made by the Governor during the month of January of such year, upon the same conditions and requirements as hereinbefore specified. Every person who shall be appointed to serve on said Board shall receive a certificate of appointment from the Secretary of the Commonwealth.

SECTION 3. The Governor shall appoint the members of said Board of Examiners from the full list of the members of the Pennsylvania State Veterinary Medical Association, which list shall, on or before the first day of January, one thousand eight hundred and ninety-five, and annually thereafter, be transmitted to the Governor under the seal and signed by the Secretary of the Association so nominating. From this list of nominees the Governor shall, during the month of January, Anno Domini one thousand eight hundred and ninety-five, appoint a Board of Veterinary Medical Examiners, to be composed exclusively of members of the same Veterinary Association. In case of failure of said Veterinary Medical Association to submit a list of members as aforesaid, the Governor shall appoint members in good standing of said Association entitled to nomination. Each one of said appointees shall have practiced veterinary medicine and surgery for at least the five years immediately preceding such appointment.

The Governor shall fill vacancies by death, or otherwise, for unexpired terms of said Examiners, from the list of said Veterinary Medical Association, and may remove any member of said Board for continued neglect of duties required by this Act, or on recommendation of the Veterinary Association, of which said members may be in affiliation, for unprofessional or dishonorable conduct.

SECTION 4. From the fees provided for by this Act the Board may pay, not to exceed said income, all proper expenses incurred by its provisions, and if any surplus above said expenses shall remain, such Examiners shall receive a reasonable remuneration for their work.

SECTION 5. The first meeting of the Examining Board shall be held on the 1st of May, one thousand eight hundred and ninety-five, suitable notice in the usual form being given, with the notice of their appointment, by the Secretary of the Commonwealth to each of the members thereof, specifying the time and place of meeting.

At the first meeting of the Board an organization shall be effected by the election, from their own membership, of a President and Secretary. For the purpose of examining applicants for license, said Board of Veterinary Medical Examiners shall hold two or more stated or special meetings in each year, due notice of which shall be made public at such times and places as they may determine. At said stated or special meetings a majority of the members of the Board shall constitute a quorum thereof, but the examination may be conducted by a committee of one or more members of the Board of Examiners duly authorized by said Board.

SECTION 6. The said Board of Veterinary Medical Examiners shall examine all diplomas as to their genuineness, and each applicant for a license shall submit to a theoretical and practical examination, said examination to be written, oral, or both; which examination, if passed successfully, shall entitle the applicant for a license permitting him to practice veterinary medicine and surgery, subject to the provisions and regulations of this Act and regulations of said Board. Such examination shall include the following subjects: Veterinary anatomy, surgery, practice of medicine, obstetrics, pathology, chemistry, veterinary diagnosis, materia medica, therapeutics, physiology, zootechnics, sanitary medicine and meat inspection.

SECTION 7. Said Board shall issue forthwith to each applicant who has passed said examination successfully, and who shall have been adjudged to be duly qualified for the practice of veterinary surgery, a license to practice the same in the State of Pennsylvania. Such license issued pursuant to this Act shall be subscribed by the officers of the Board of Veterinary Medical Examiners. It shall also have affixed to it, by the person authorized to affix the same, the seal of this Commonwealth.

Before said license shall be issued it shall be recorded in a book to be kept in the office which such Board may establish for the purpose of carrying out the provisions of this Act, and the number of this book and the page therein containing said recorded copy shall be noted upon the face of said license.

Such records shall be open to public inspection with proper restrictions as to their safe keeping, and in all legal proceedings shall have the same weight as evidence that is given to the conveyance of land.

SECTION 8. From and after the first day of July, Anno Domini one thousand eight hundred and ninety-five, any person not heretofore authorized to practice veterinary medicine and surgery in this State, and desiring to enter upon such practice, may deliver to the Secretary of the Veterinary Medical Board, upon the payment of a fee of twenty-five dollars ($25.00), a written application for license, together with satisfactory proof that the applicant is more than twenty-one years of age, is of good moral character, has obtained a competent common school education, and has received a diploma conferring the degree of veterinary medicine from some legally incorporated veterinary college of the United States, or a diploma, or license, conferring the full right to practice all the branches of veterinary surgery in some foreign country; applicants who have received their degree in veterinary medicine, after the first day of July, one thousand eight hundred and ninety-five, must have pursued the study of veterinary medicine for at least three years, including three regular courses of lectures, in different years, in some legally incorporated veterinary college or colleges, prior to the granting of said diploma or foreign license. Such proof shall be made, if required, upon affidavit. Upon making the said payment and proof, the Examining Board, if satisfied with the same, shall issue to such applicant an order for examination. In case of failure at any such examination, the candidate, after the expiration of six months, and within two years, shall have the privilege of a second examination by the same Board to which application was first made, without the payment of an additional fee; and it is further provided, that applicants examined and licensed by State Boards of Veterinary Medical Examiners of other States, on payment of a fee of ten dollars ($10.00) to the Examining Board, and on filing in the office of said Board a copy of said license, certified by the affidavit of the President or Secretary of the Board of such other State, showing also that the standard of requirements adopted by that State Board of Veterinary Medical Examiners is substantially the same as is provided by sections four, five and six of this Act, shall, without further examination, receive a license conferring upon the holder thereof all the rights and privileges provided by sections eight and nine of this Act.

SECTION 9. From and after the first day of March, Anno Domini one thousand eight hundred and ninety-five, no person shall enter upon the practice of veterinary medicine and surgery in the State of Pennsylvania unless he has complied with the provisions of this Act, and shall have exhibited to the prothonotary of the Court of Common Pleas of the county in which he desires to practice veterinary medicine and surgery a license duly granted to him as hereinbefore provided, whereupon he shall be entitled, upon the payment of one dollar ($1.00), to be duly registered in the office of the prothonotary of the Court of Common Pleas in the said county, and any person violating any of the provisions of this Act shall be guilty of a misdemeanor, and upon conviction thereof in the Court of Quarter Sessions of the county wherein the offense shall have been committed, shall pay a fine of not more than two hundred dollars ($200.00) for each offense.

SECTION 10. Nothing in this Act shall be construed to interfere with or punish commissioned veterinarians in the United States Army, or any lawfully qualified veterinarian residing in other States or countries, meeting registered veterinarians of this State in consultation, or any veterinarian residing on the border of a neighboring State, and duly authorized under the laws thereof, to practice veterinary medicine and surgery therein, whose practice extends into the limits of this State: provided, that such practitioner shall not open an office or appoint a place to meet patients or receive calls within the limits of Pennsylvania. And nothing in this Act shall be construed to prohibit the practice of veterinary medicine and surgery, within this Commonwealth, by any practitioner who shall have been duly registered before the first day of March, Anno Domini one thousand eight hundred and ninety-five, and one such registry shall be sufficient warrant to practice veterinary medicine and surgery in any county in this Commonwealth.

SECTION 11. Said Board shall be the prosecutor in all cases under this Act, and such fine may be imposed by any Justice of the Peace of any borough, city or county where such offense may have been committed.

SECTION 12. All Acts or parts of Acts of Assembly inconsistent herewith shall be and are hereby repealed.

